# Psychological Crisis Intervention for COVID-19 Lockdown Stress in Patients With Type 1 Diabetes Mellitus: Survey Study and Qualitative Analysis

**DOI:** 10.2196/28097

**Published:** 2021-06-30

**Authors:** Katarzyna Cyranka, Dominika Dudek, Maciej Tadeusz Małecki, Bartłomiej Matejko, Tomasz Klupa

**Affiliations:** 1 Department of Psychiatry Jagiellonian University Medical College Krakow Poland; 2 Department of Metabolic Diseases Jagiellonian University Medical College Krakow Poland

**Keywords:** COVID-19, pandemic, type 1 diabetes mellitus, crisis psychological intervention, anxiety, stress, psychosomatics, diabetes, type 1, psychology, crisis situation, intervention

## Abstract

**Background:**

The COVID-19 pandemic has challenged the worldviews of most people. Social isolation after the COVID-19 lockdown has not only led to economic difficulties but also resulted in adverse psychological reactions. As in most countries, including Poland, this situation has been very challenging for patients with type 1 diabetes mellitus (T1DM). In Poland, a crisis intervention team for patients with T1DM was established. The goal of the team was to provide psychological support for these patients, if needed, and to present information concerning how these patients may obtain medical consultations and prescriptions.

**Objective:**

We aimed to analyze the psychological parameters and main emotional reactions of patients with T1DM during the COVID-19 lockdown.

**Methods:**

An email with information concerning the possibility of having a web-based consultation with psychologists and psychiatrists and an attached set of psychological tests was sent to all patients with T1DM who were under the care of an outpatient diabetes clinic. The consultations were performed by licensed clinical psychologists and psychologists. This study was approved by the Bioethics Committee of the Jagiellonian University in Krakow, Poland.

**Results:**

The patients who decided to use psychological support had statistically higher levels of anxiety (state *P*=.043; trait *P*=.022), stress (*P*=.001) than those of patients from the group who did not seek support.

**Conclusions:**

The presented intervention team may be perceived as an example of important and successful cooperation and communication between specialists of different fields of medicine (diabetology, psychiatry, and psychology) in a crisis situation.

## Introduction

### Background

The COVID-19 pandemic, with its subsequent mental health consequences, has challenged the worldviews of most people. Social isolation after the COVID-19 lockdown has not only led to economic difficulties but also resulted in adverse psychological reactions. People’s most common reactions when faced with such life-threatening circumstances are stress, anxiety, and depression [1]. People with diabetes and other comorbid conditions, such as asthma, heart failure, raised serum creatinine levels, and an age of >65 years, have turned out to be at higher risk of SARS-CoV-2 infection complications and the emotional burden related to such complications [2]. Regardless of the potential spread of COVID-19, as with influenza-related mortality, diabetes is an important risk factor for adverse outcomes [3]. With the medical focus being largely centered on COVID-19, the clinical support needed by patients living with noncommunicable diseases, including type 1 diabetes mellitus (T1DM), has been severely affected. Treatment delays, the discontinuation of routine care, a lack of services, and the uncertainty surrounding medicine availability have left these patients more at risk than ever before. A survey of 155 countries by the World Health Organization reported the dramatic curtailment of health services for patients living with noncommunicable diseases during the COVID-19 pandemic. It was reported that diabetes treatment has been partially or completely disrupted in 49% of the countries surveyed [4]. Glycemic control is important for any patient with COVID-19. Although there is limited data about the association between blood glucose levels and the disease course of COVID-19 at present, data regarding other infections, such as SARS (severe acute respiratory syndrome) and influenza H1N1 infection, have shown that patients with poor glycemic control have an increased risk of complications and death [5-7]. In many countries. organizing proper care for people with T1DM in a pandemic situation became a pressing issue [8]. In the United Kingdom, the National Diabetes Inpatient COVID Response Group was formed at the end of March 2020 to support the provision of diabetes inpatient care during the COVID-19 pandemic. It was formed in response to two emerging needs. The first was to ensure that basic diabetes services were secured and maintained at a time when there was a call for redeployment to support the need for general medical expertise across secondary care services. The second was to provide simple, safe diabetes guidelines for use by specialists and nonspecialists treating inpatients with confirmed or suspected SARS-CoV-2 infection [9]. As in most countries, including Poland, the COVID-19 pandemic has been very challenging for patients with T1DM, especially during the first 2 months of the pandemic lockdown. In the Malopolska region, around 600 patients with T1DM found themselves in a difficult situation due to the transformation of the University Hospital in Krakow into an infectious diseases center for patients with COVID-19. Thus, some diabetologists who were working on a regular basis in the Outpatient Diabetology Clinic have been transferred to work with patients with COVID-19 and cut off from their patients with T1DM. In Poland, a crisis intervention team for patients with T1DM was established. The goal of the team was to provide psychological support for these patients, if needed, and to present information concerning how these patients may obtain medical consultations and prescriptions.

### Aim of This Study

This study presents data from telephone consultations that were carried out by the crisis intervention team between April 14 and May 11, 2020, and subsequent interventions.

## Methods

### Study Design

An email with information concerning the possibility of having a web-based (email, Skype, or telephone) consultation with psychologists and psychiatrists and an attached set of psychological tests was sent to 473 patients with T1DM who were under the care of an outpatient diabetes clinic. Of all the individuals who received the email, 50 patients filled in the set of questionnaires and reported their mental condition, and 34 patients did not need psychological support. However, by means of email or telephone contact, these 34 patients asked questions concerning medical issues (eg, how they may contact their diabetologist, how they may obtain prescriptions for insulin, etc), and they were given proper information. In total, 20 patients decided to attend telephone-based psychological consultations. There were 24 consultations conducted from April 14 to May 11, 2020. The average consultation time was 45 minutes; the shortest consultation lasted 20 minutes, and the longest consultation lasted 1.5 hours.

The crisis intervention team included the following: 2 psychiatrists (one was a professor in psychiatry and the head of the Department of Psychiatry, and the other one was also a psychotherapist), 2 clinical psychologists (one with a specialization in psychosomatics and one with a specialization in adult psychopathology; the latter was also a certificated psychotherapist), 1 certificated supervisor of psychotherapy, and 1 professor and regional consultant in diabetology

This study was approved by the Bioethics Committee of the Jagiellonian University in Krakow, Poland, on January 4, 2020 (approval number: 1072.6120.78.2020).

The set of psychological tools included the following:

1. The Coping Inventory for Stressful Situations (CISS)—a 4-factor model of humans coping with adversity developed by Endler et al [10]. Their construct differentiates the following three types of coping: task-oriented coping, emotion-oriented coping, and avoidant-oriented coping.
2. The State-Trait Anxiety Inventory (STAI) by Spielberger et al [11]—a commonly used measure of trait and state anxiety.
3. The Perceived Stress Scale-10 (PSS-10) designed by Cohen et al [12]—the most widely used psychological instrument for measuring the perception of stress.
4. The General Health Questionnaire-30 (GHQ-30)—a screening tool for identifying minor psychiatric disorders in the general population and within community or nonpsychiatric clinical settings, such as primary care settings or general medical outpatient care settings. It assesses a respondent’s current state and asks if that state differs from their usual state [13].

### Studied Group

In total, 20 persons—7 men and 13 women—contacted a psychologist. All the consulted persons were patients of the University Hospital in Krakow undergoing insulin pump T1DM treatment.

During the first period of lockdown, 1 person needed 3 consultations (1 woman), 2 people had 2 consultations, and 17 people had single consultations. The average age of the consulted persons was 25 years. Further, 6 patients who asked for a crisis consultation were currently undergoing or had previously undergone psychotherapy.

The data on the underlying psychiatric diagnoses at the time of this study were not complete; 2 patients indicated having depression, 3 said that they had anxiety disorder, and 1 admitted having eating disorder. As a result of the consultations, of the 20 patients consulted, 7 were diagnosed with anxiety disorder (chapter F4 of the International Classification of Diseases, Tenth Revision [ICD-10]) and depression (chapter F3 of the ICD-10), 5 were diagnosed with personality disorder (chapter F6 of the ICD-10), and 2 were diagnosed with eating disorder (chapter F5 of the ICD-10). The diagnoses were based on the ICD-10. Additionally, in 11 patients, adjustment disorder was diagnosed and directly connected to the pandemic situation as a comorbid or main diagnosis. At the consultation stage, the patients claimed that their basic need was to discuss their situations with a psychologist. However, during the later stages of the diagnostic process, 7 patients were referred to psychiatrists for consultation and 5 patients were offered pharmacotherapy. There was no additional survey conducted at the end of this study, but all patients who required further diagnostic procedures and treatment were provided with these benefits.

### Data Availability

The data that support the findings of this study are available from the corresponding author, KC, upon reasonable request.

## Results

### Consultation Topics

The main problems that were discussed with the patients with T1DM during telephone intervention during the pandemic lockdown are categorized and presented in Textbox 1.

Of the 20 patients who attended the telephone consultations, 10 submitted their set of questionnaires, and the rest decided to stay anonymous or refused to fill in the questionnaires for various reason.

We decided to carry out statistical analyses for the group of 10 patients who submitted their questionnaires, bearing in mind that they only made up half of the group that underwent psychological intervention, and we compared their results to the results of patients with T1DM who did not need intervention but also filled in the questionnaire screening tools during the pandemic lockdown.

Statistical analyses were carried out using the IBM SPSS Statistics 25 package (IBM Corporation). *U* Mann-Whitney tests were performed. The classical threshold α value of .05 was used as the level of significance.

**Main topics issued by the patients during crisis intervention and their descriptions.**

**Fear of SARS-CoV-2 infection**

- The patients reported high levels of anxiety.
- Worried that they may become infected with SARS-CoV-2 and that they may die or experience complications of diabetes after being infected, 5 patients described nightmares related to the COVID-19 pandemic.

**Feeling depressed**

- After a few weeks of lockdown, 13/20 (65%) of the patients who asked for a consultation reported a feeling of sadness, a lack of energy, depressiveness, and a feeling of helplessness.
- Some patients indicated that they were sad because they lost control over their diabetes as a result of an emotional crisis and lockdown-related changes in their daily routine.

**Fear of insufficient medical care**

- In total, 19/20 (95%) of the patients admitted that they experienced some level of stress, sadness, or anger because of limited access to the attending physician, especially patients who had their planned visits postponed or cancelled.

**The fear that the attending physician will get hurt or that he will become sick with COVID-19**

- As some of the patients had information regarding their attending physician being transferred to work with patients with COVID-19, they expressed fears that he may become infected and that he may not come back to his previous work.
- In total, 3/20 (15%) patients were angry that the hospital “preferred” patients with COVID-19 and took their diabetologist away.
- In total, 4/20 (20%) patients hoped that by conducting the crisis telephone calls, they would be able to send supporting words to their doctors. The patients wished their doctors strength and all the best, which reflected good patient-diabetologist relations and the patients’ positive attitudes toward their diabetologists.

**Disorganization of everyday functioning**

- In total, 16/20 (80%) of the patients who used the telephone interventions admitted to having serious problems with their daily functioning, which intensively affected their type 1 diabetes mellitus control. The dysfunctions included diet modifications, problems with regularly exercising, and financial and work difficulties that resulted in changes in work routine and lowered income (a few patients feared that they would not be able to use an insulin pump because of the loss of work).

**The fear that insulin will not be available**

- In total, 3/20 (15%) of the patients had a strong fear that insulin would not be available and that they would die as a result. After longer conversations, it turned out that the three patients had family trauma concerning Siberia or Auschwitz experiences, which could be interpreted as transmitted family war experiences related to a high level of fear concerning a lack of supplies.

**Family conflicts due to being together for a long time**

- In total, 14/20 (70%) of the consulted patients reported some family tensions or conflicts related to the need to spent a lot of time together in an unexpected lockdown situation.
- The patients connected their stress with worse glycemic control.

**Need to describe how the patient is coping with an epidemic (to gain reinforcement)**

- In total, 5 patients decided to attend the consultations to make sure that they were coping well and to describe how they cope with everyday lockdown challenges concerning type 1 diabetes mellitus and how they support other patients with the disease. They also searched for reinforcement and the possibility of not being alone during their everyday challenges.

**Worsening of previously present symptoms of mental disorders**

- In total, 5 of the consulted patients turned out to have comorbidities to a type 1 diabetes mellitus psychiatric diagnosis. Further, they previously underwent psychological or psychiatric treatment. Some of them had limited access to their therapist and felt the need to discuss their current state. Others were still in contact with their therapist, but they felt that they needed additional crisis consultations.
- All patients who needed further psychotherapy or psychiatric consultations were taken care of, and such help was provided.

### Level of Anxiety in the Examined Groups Measured With the STAI

In the first step, we checked whether the levels of state and trait anxiety varied between the studied groups. As shown in Table 1, there were statistically significant differences in terms of both these variables. A higher level of anxiety was recorded for the group of patients who asked for psychological intervention. The strength of the observed effects was moderately high (Table 1).

**Table 1:** Level of anxiety in the examined groups.

Anxiety type	Scores of patients with T1DM^a^ who asked for psychological intervention (n=10), mean (SD)	Scores of patients with T1DM who did not ask for psychological intervention (n=39), mean (SD)	*U*	Z	*P* value	*r*
State anxiety	47.80 (11.29)	40.08 (9.36)	113.5	−2.02	.04	0.29
Trait anxiety	47.20 (8.09)	40.46 (8.09)	102.5	−2.30	.02	0.33

^a^T1DM: type 1 diabetes mellitus.

### Level of Stress in the Examined Groups Measured With the PSS-10

In the second step, we checked whether the levels of experienced stress were different between the studied groups. As shown in Table 2, a statistically significant difference was noted. A higher level of stress was recorded for the group of patients who needed crisis intervention. The strength of the observed effect was high (Table 2).

**Table 2:** Level of stress in the examined groups.

Type of stress	Scores of patients with T1DM^a^ who asked for psychological intervention (n=10), mean (SD)	Scores of patients with T1DM who did not ask for psychological intervention (n=39), mean (SD)	*U*	Z	*P* value	*r*
Level of perceived stress	23.60 (5.42)	15.44 (6.30)	62.0	−3.30	.001	0.47

^a^T1DM: type 1 diabetes mellitus.

### Styles of Coping With Stress in the Examined Groups Measured With the CISS

Table 3 shows that there was 1 statistically significant difference in coping strategies between the patients with T1DM who asked for intervention and those who did not. In the intervention group, a higher emotion-focused coping style level was noted. The differences in task-oriented coping style (*P*=.27), avoidant-oriented coping style (*P*=.35), distraction seeking (*P*=.42), and social diversion (*P*=.053) levels between the 2 groups were not statistically significant.

**Table 3:** Styles of coping with stress in the examined groups.

Style	Scores of patients with T1DM^a^ who asked for psychological intervention (n=10), mean (SD)	Scores of patients with T1DM who did not ask for psychological intervention (n=39), mean (SD)	*U*	Z	*P* value	*r*
Task-oriented style	53.40 (10.34)	56.67 (9.08)	150.5	−1.11	.27	0.16
Emotion-oriented style	49.30 (6.86)	38.23 (10.51)	60.0	−3.36	.001	0.48
Avoidant-oriented style	40.00 (5.87)	42.54 (8.64)	157.0	−0.94	.35	0.13
Distraction seeking	18.30 (3.83)	17.56 (5.68)	162.5	−0.81	.42	0.12
Social diversion	13.90 (4.12)	16.92 (3.92)	117.5	−1.93	.053	0.28

^a^T1DM: type 1 diabetes mellitus.

### General Mental Health Condition Measured With the GHQ-30

Finally, we checked whether the level of general mental health, which was measured as both a condition and a feature of the examined patients, varied between the studied groups. As can be seen in Table 4, there were statistically significant differences in all three analyzed variables. Higher scale scores were noted for the group that needed crisis intervention. The strength of the observed effects was very large for the anxiety and depression scale scores and large for the other two variables (Table 4).

**Table 4:** General mental health in the examined groups.

Mental health variables	Scores of patients with T1DM^a^ who asked for psychological intervention (n=10), mean (SD)	Scores of patients with T1DM who did not ask for psychological intervention (n=39), mean (SD)	*U*	Z	*P* value	*r*
Fear and depression	2.58 (0.30)	1.85 (0.41)	20.0	−4.36	<.001	0.62
Interpersonal relations	2.38 (0.41)	1.97 (0.39)	97.0	−2.51	.01	0.36
General functioning	2.76 (0.53)	2.30 (0.25)	87.0	−2.73	.006	0.39

^a^T1DM: type 1 diabetes mellitus.

## Discussion

### Principal Findings

A wide array of evidence—ranging from epidemiological evidence to animal model evidence—points toward the role of psychological stressors in T1DM pathogenesis. Various mechanisms have been proposed, including the hypothalamic-pituitary-adrenal axis, the influence of the nervous system on immune cells, and insulin resistance [14]. Studies have also indicated that general psychological stress plays a role in the glycemic control of individuals with T1DM [15]. A study of 1396 people with diabetes by Joensen et al [16] showed that worries related to the COVID-19 pandemic are highly prevalent. The participants were most frequently worried about “being overly affected due to diabetes if infected with COVID-19” (56%), were worried that “people with diabetes are characterized as a risk group” (39%), and were worried about “not being able to manage diabetes if infected with COVID-19” (28%). Further, 25% of the participants experienced diabetes distress at the beginning of the COVID-19 pandemic [16]. With regard to young people and adolescents, stress and social isolation may affect brain health and development. A UK survey that was conducted during the COVID‐19 pandemic found that 83% (1752/2111) of young people with a history of mental health needs felt that their mental health was worse. Further, one-quarter of participants (5/20, 25%) were no longer able to access their usual mental health support [17].

The 2020 pandemic lockdown situation has turned out to be a globally recognized stressor, especially for people with chronic diseases. For individuals with T1DM, this situation has resulted in a multifaceted threat related to their limited access to their attending physician, changes in the everyday organization of life, a sense of threat related to the possibility of being infected and experiencing complications, and many other detriments. Health care providers face the new challenges of providing optimal medical and psychological care for this group of patients and taking into account the difficulties related to the epidemiological situation. University Hospital in Krakow took up this challenge through the collaboration between the Department and Clinic of Metabolic Diseases and the Department of Psychiatry and created a crisis intervention team for patients with T1DM that was available 24 hours per day during the first stage of the lockdown. The patients who decided to engage with this psychological support team reported a set of specific emotional problems and difficulties that, in their opinion, had a major impact on their glycemic control during this period. The patients presented a wide range of problematic issues concerning various aspects of their lives; however, all of these issues were related to their diabetes control. The possibility of using psychological support was a form of intervention that did not replace diabetological consultation but helped the patients deal with the stressful situation and thus release their tension and improve their feelings of control over the disease. Additionally, the consultations were a source of information on how to obtain medical support, if needed. In the group of patients who decided to use psychological support, 10 persons filled in a set of questionnaires that assessed their mental condition. Their test results indicated high levels of stress, anxiety, and general mental psychopathology in comparison to the patients with T1DM who did not ask for psychological support during the lockdown. Further, it was found that this group of patients was less task oriented and that they reacted very emotionally to the stressful COVID-19 pandemic situation.

Although this study made important observations, it also has some limitations. As the crisis intervention team was designed under the specific conditions of the first stage of pandemic, the main aim was to provide support to the patients as soon as possible. Thus, we did not collect sociodemographic data from all of the patients. Additionally, some of the patients who wanted consultations for their situations did not send their set of questionnaires, as they wished to keep their anonymity. Therefore, we could not analyze the questionnaire results of the whole intervention group. Given these conditions, we also did not focus on analyzing the correlation between the metabolic parameters of the patients and their mental health test results. As the work of the crisis intervention team has continued, we were able to include data on the diagnostic process.

Although the results cannot be generalized due to the small sample, they may suggest that there is a group of patients with T1DM who need regular, special psychological attention due to psychological conditions, which may influence their glycemic control. Apart from their reactions to stress, some patients turned out to have comorbid personality disorder or eating disorder. These disorders develop over time, with some developing from early childhood. Thus, we assumed that these disorders were present before the pandemic lockdown and that this stressful situation was, for some patients, a moment of decompensation. This observation requires further analysis and attention.

### Conclusion

The presented intervention team may be perceived as an example of important and successful cooperation and communication between specialists of different fields of medicine (diabetology, psychiatry, and psychology) in a crisis situation. Although consultations were conducted for a small group of patients, the content of the consultations indicated the legitimacy of and the need for such cooperation on a regular basis, as the mental health of patients with T1DM and their stress management are two of the essential factors in achieving glycemic control.

## Abbreviations

CISS: Coping Inventory for Stressful Situations
GHQ-30: General Health Questionnaire-30
ICD-10: International Classification of Diseases, Tenth Revision
PSS-10: Perceived Stress Scale-10
SARS: severe acute respiratory syndrome
STAI: State-Trait Anxiety Inventory
T1DM: type 1 diabetes mellitus
